# Suprascapular Nerve Pulsed Radiofrequency for Chronic Shoulder Pain in a Pediatric Patient

**DOI:** 10.1155/2020/5709421

**Published:** 2020-06-29

**Authors:** Federico Cristiani, Maria A. Hernandez

**Affiliations:** ^1^Department of Anesthesiology, Pereira Rossell Pediatric Hospital, Montevideo, Uruguay; ^2^Department of Anesthesiology and Perioperative Medicine, University of Pittsburgh Medical Center, Pittsburgh, USA

## Abstract

Pulsed radiofrequency of the suprascapular nerve has shown efficacy in adults with chronic shoulder pain, but its use in pediatrics is relatively new. We present a case of a successful use of pulsed radiofrequency to treat refractory chronic shoulder pain in an adolescent patient. *Case Report*. We present a 53 kg, 14-year-old female, with a medical history of septic arthritis of the left shoulder within the first month of life leading to persisting pain during childhood. She presented with a complaint of progressive pain starting at age 12, refractory to analgesics including opioids and intra-articular injection of local anesthetic and steroids. After pulsed radiofrequency of the suprascapular nerve, significant pain relief and improvement of the range of motion was obtained. These results were maintained at the 1-, 3-, and 6-month follow-up visits with the pain being reported as mild and manageable with nonsteroidal anti-inflammatory drugs. *Conclusion*. There is limited data today of the use of pulsed radiofrequency for pediatric chronic pain. We successfully used this intervention in a 14-year-old patient with chronic shoulder pain extrapolating from adult experience and as a last resort after all other treatments had failed.

## 1. Introduction

Shoulder pain is relatively common in the general population and may result from an injury, inflammatory conditions, or a degenerative process. The most common causes are related to rotator cuff disorders, glenohumeral and acromioclavicular capsulitis, or arthritis and joint instability [1, 2]. Most patients with chronic shoulder pain respond well to physical therapy, nonsteroidal anti-inflammatory drugs (NSAIDs), and activity modification. Injection of a local anesthetic with steroids is also effective to alleviate inflammation and to allow passive and active physical therapy [1, 3–5]. When the pain is intractable, a trial of pulsed radiofrequency (PRF) of the suprascapular nerve may be indicated as a means to delay or avoid surgery [6–8].

Pulsed radiofrequency was developed as a modification of continuous radiofrequency. In the latter, a high-frequency alternating current is applied to elevate the temperature above 45–50°C to produce nonselective coagulative necrosis of the nociceptive fibers. In PRF, on the other hand, high-voltage short bursts of current (20 ms) are followed by silent phases (480 ms) allowing diffusion of heat, maintaining the temperature in the target tissue below 42°C with the intention to produce prolonged “stunning” of nerve fibers without irreversible cell damage [8–11].

Pulsed radiofrequency (PRF) of the suprascapular nerve has shown efficacy in adults with chronic shoulder pain [7, 8, 10–13], but there are no reports of its use in children. We present a case were an ultrasound (US) guided PRF of the suprascapular nerve successfully alleviated refractory chronic shoulder pain in a pediatric patient.

## 2. Case Report

**Figure 1:**
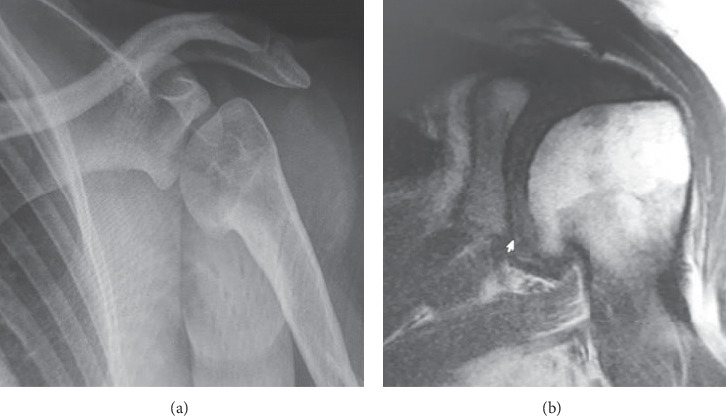
Radiography (a) and magnetic resonance images (b) showing the loss of sphericity associated with osteophytes in the inferior and internal area of the humeral head.

Informed consent was obtained from the patient and parents for the procedure and later for publication. A 53 kg, 14-year-old female, with a past medical history of septic arthritis of the left shoulder within the first month of life, presented with a complaint of progressive pain since age 12. Her pain had been refractory to analgesics including opioids, physical therapy, and intra-articular injection of local anesthetic and steroids for the past two years. The patient was referred to the pain clinic after a trial of pregabalin and tramadol was unhelpful and 2 intra-articular injections of steroids and local anesthetics failed to provide analgesia. The patient presented with severe pain (numeric rating scale ≥7/10), localized in the posterior and superior area of the glenohumeral joint, exacerbated by passive and active abduction of the arm of more than 90 degrees. No swelling, periarticular redness, or joint deformity were present. The pain appeared to be somatic in nature, with no neuropathic features such as allodynia, paresthesia, burning sensation, or changes of the skin color. X-ray films and magnetic resonance images showed signs compatible with postinfectious arthritis including osteophytes in the inferior and internal area of the humeral head (Figure 1).

We performed diagnostic ultrasound-guided injection of the suprascapular nerve with bupivacaine 0.125% 6 ml and dexamethasone 4 mg resulting in complete pain relief for 15 days, returning to baseline severe pain scores afterwards. Given the favorable result of the diagnostic injection we decided to perform PRF of the suprascapular nerve.

**Figure 2:**
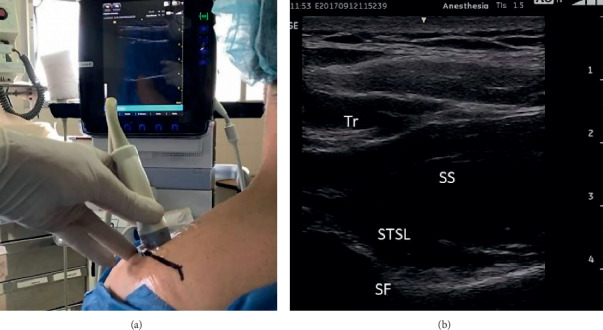
US-guided suprascapular nerve localization. (a). Position of the transducer. (b) Tr: trapezius muscle, SS: supraspinatus muscle, STSL: superior transverse scapular ligament, and SF: suprascapular fossa.

The procedure was performed in the operating room with the patient seated and monitored by an anesthesiologist. Sedation was not required, and the patient tolerated the procedure well. A high-frequency linear transducer (10–15 Hz) was placed parallel and cephalad to the spine of the scapula (Figure 2(a)). The probe was moved from cephalad to caudad until the trapezius and supraspinatus muscles were identified. The suprascapular nerve was identified as a hyperechogenic structure in the suprascapular fossa deep to the superior transverse scapular ligament (Figure 2(b)). After skin infiltration with 3 ml of lidocaine 1%, a 10 cm radiofrequency needle, with a 10 mm active tip, was inserted in plane with the ultrasound beam and advanced to reach the proximity to the nerve. A nerve stimulator was used as an additional nerve-finding modality. Sensitive stimulation (50–100 Hz at 0.4 Volts) elicited paresthesia in the posterior aspect of the shoulder. Motor stimulation (2 Hz at 0.5 V) elicited muscle contraction of the supraspinatus and infraspinatus muscles. After administration of 2 ml of lidocaine 1%, PRF was applied to the suprascapular nerve at 45 V 42°C, in 2 cycles of 120 seconds. The procedure was well-tolerated. After 10 mins, the patient related complete resolution of the pain and was able to perform a full range of shoulder motions. She was discharged home with the instructions to use NSAIDs as needed. The patient presented for follow-up appointments at 1, 3, and 6 months and reported only mild intermittent pain that responded to NSAIDs.

## 3. Discussion

This case report suggests that PRF may be considered for the management of chronic shoulder pain in children when conservative treatment and intra-articular injections of local anesthetic and steroids fail. In our case, a successful response was evidenced by an increased range of motion and improvement of pain scores, consistent with the adult literature [7, 9–16]. After a single treatment, the pain was manageable with minimal analgesic requirements at the 6-month follow-up.

The suprascapular nerve originates from the superior trunk of the brachial plexus. It provides 70% of the sensory innervation of the shoulder supplying the posterior and superior aspect of the glenohumeral joint, the joint capsule, and the acromioclavicular joint. It also provides motor innervation to the supra- and infraspinatus muscles [17].

There are concerns about using radiofrequency in children because of the potential risks of nerve injury that may cause a permanent loss of function at an early age. However, several studies in adults have reported the safety of this technique making this treatment attractive. The injury caused by PRF has a selective effect on small sensory fibers, A-*δ* and C-fibers [8–11]. It has been postulated that RF causes rectification of Na^+^ and K^+^ channels leading to a depolarization state which inhibits neural cells from reaching the action potential threshold in response to stimuli, therefore decreasing the neural transmission of painful sensations and ectopic discharges [18–21].

Ultrasound guidance offers the advantage of real-time imaging of the nerve and needle guidance, thus decreasing the potential for pneumothorax and vascular puncture, without the need of radiation exposure for the patients and personnel.

The decision to perform this procedure despite limited previous experience in children was taken considering the low risk potential and the possible benefits of avoiding surgery in view of the failed previous therapies. Procedural interventions in pediatrics are generally performed with scarce evidence and based on adult experience. The positive results of this case report suggest that PRF may be effective in children and could be considered if the potential benefits outweigh the risks when noninvasive treatments fail.
